# Supplementary material for: FERN – a Java framework for stochastic simulation and evaluation of reaction networks
Source: BMC Bioinformatics. 2008 Aug 29;9:356. doi: 10.1186/1471-2105-9-356 (PMC2553347; doi:10.1186/1471-2105-9-356)
Supplement: Additional file 1 — FERN distribution, Version 1.3. This archive contains the FERN source code and binaries as well as documentation and example models in FernML and SBML. [file 1471-2105-9-356-S1.zip › fern/doc/javadoc/serialized-form.html]

Serialized Form


---


|  |  |  |
| --- | --- | --- |
| **Overview** \| Package \| Class \| Use \| **Tree** \| **Deprecated** \| **Index** \| **Help** | |  |
| PREV   NEXT | **FRAMES**    **NO FRAMES**     **All Classes** |  |


---


# Serialized Form


---

**Package** **fern.analysis**

**Class fern.analysis.IntQueue extends IntArrayList implements Serializable**

**serialVersionUID:** 1L

**Class fern.analysis.IntStack extends IntArrayList implements Serializable**

**serialVersionUID:** 1L

---

**Package** **fern.cellDesigner.ui**

**Class fern.cellDesigner.ui.ExtendedPane extends JPanel implements Serializable**

**serialVersionUID:** 1L

**Serialized Fields**

### main

```
MainFrame main
```

---

### nodeTypePicker

```
JComboBox nodeTypePicker
```

---

### reactionPicker

```
JComboBox reactionPicker
```

---

### speciesPicker

```
JComboBox speciesPicker
```

---

### initialAmountPicker

```
JComboBox initialAmountPicker
```

---

### reactioncoefficientPicker

```
JComboBox reactioncoefficientPicker
```

---

### eps

```
JTextField eps
```

---

### nc

```
JTextField nc
```

---

### thresh

```
JTextField thresh
```

---

### num

```
JTextField num
```

---

### notList

```
JList notList
```

---

### trendList

```
JList trendList
```

**Class fern.cellDesigner.ui.MainFrame extends JFrame implements Serializable**

**serialVersionUID:** 1L

**Serialized Fields**

### network

```
CellDesignerNetworkWrapper network
```

---

### overview

```
OverviewPane overview
```

---

### extended

```
ExtendedPane extended
```

---

### startStopButton

```
JButton startStopButton
```

---

### progress

```
JProgressBar progress
```

---

### startSimulation

```
fern.cellDesigner.ui.MainFrame.SimulationAction startSimulation
```

---

### plugin

```
FernCellDesignerPlugin plugin
```

**Class fern.cellDesigner.ui.OverviewPane extends JPanel implements Serializable**

**serialVersionUID:** 1L

**Serialized Fields**

### main

```
MainFrame main
```

---

### networkStatus

```
JLabel networkStatus
```

---

### message

```
JTextArea message
```

---

### simulator

```
JComboBox simulator
```

---

### time

```
JTextField time
```

---

### runs

```
JTextField runs
```

---

**Package** **fern.cytoscape**

**Class fern.cytoscape.CytoscapeVisualizer.ShowMainFrameAction extends cytoscape.util.CytoscapeAction implements Serializable**

**serialVersionUID:** 1L

**Class fern.cytoscape.CytoscapeVisualizer.SimulationAction extends cytoscape.util.CytoscapeAction implements Serializable**

**serialVersionUID:** 1L

---

**Package** **fern.cytoscape.ui**

**Class fern.cytoscape.ui.ColorPicker extends JDialog implements Serializable**

**serialVersionUID:** 1L

**Serialized Fields**

### reactionColor

```
fern.cytoscape.ui.ColorPicker.ColorField reactionColor
```

---

### amountBottomColor

```
fern.cytoscape.ui.ColorPicker.ColorField amountBottomColor
```

---

### amountTopColor

```
fern.cytoscape.ui.ColorPicker.ColorField amountTopColor
```

---

### preview

```
fern.cytoscape.ui.ColorPicker.PreviewField preview
```

---

### scaleType

```
JComboBox scaleType
```

---

### scaleMax

```
JTextField scaleMax
```

---

### colcalc

```
ColorCalculator colcalc
```

---

### action

```
fern.cytoscape.ui.ColorPicker.PreviewChangeAction action
```

**Class fern.cytoscape.ui.ExtendedPane extends JPanel implements Serializable**

**serialVersionUID:** 1L

**Serialized Fields**

### main

```
MainFrame main
```

---

### nodeTypePicker

```
JComboBox nodeTypePicker
```

---

### reactionPicker

```
JComboBox reactionPicker
```

---

### speciesPicker

```
JComboBox speciesPicker
```

---

### initialAmountPicker

```
JComboBox initialAmountPicker
```

---

### reactioncoefficientPicker

```
JComboBox reactioncoefficientPicker
```

---

### eps

```
JTextField eps
```

---

### nc

```
JTextField nc
```

---

### thresh

```
JTextField thresh
```

---

### num

```
JTextField num
```

---

### notList

```
JList notList
```

---

### trendList

```
JList trendList
```

**Class fern.cytoscape.ui.MainFrame extends JFrame implements Serializable**

**serialVersionUID:** 1L

**Serialized Fields**

### network

```
CytoscapeNetworkWrapper network
```

---

### overview

```
OverviewPane overview
```

---

### extended

```
ExtendedPane extended
```

---

### startStopButton

```
JButton startStopButton
```

---

### progress

```
JProgressBar progress
```

---

### startSimulation

```
fern.cytoscape.ui.MainFrame.SimulationAction startSimulation
```

---

### recentNetworkChecker

```
NetworkChecker recentNetworkChecker
```

---

### style

```
FernVisualStyle style
```

**Class fern.cytoscape.ui.OverviewPane extends JPanel implements Serializable**

**serialVersionUID:** 1L

**Serialized Fields**

### main

```
MainFrame main
```

---

### networkStatus

```
JLabel networkStatus
```

---

### message

```
JTextArea message
```

---

### simulator

```
JComboBox simulator
```

---

### time

```
JTextField time
```

---

### realTime

```
JCheckBox realTime
```

---

### runs

```
JTextField runs
```

---

### visu

```
JCheckBox visu
```

---

**Package** **fern.network**

**Class fern.network.FeatureNotSupportedException extends Exception implements Serializable**

**serialVersionUID:** 1L

---

**Package** **fern.tools.gnuplot**

**Class fern.tools.gnuplot.GnuPlot extends JFrame implements Serializable**

**serialVersionUID:** 1L

**Serialized Fields**

### img

```
BufferedImage img
```

---

### commands

```
List<E> commands
```

---

### axes

```
List<E> axes
```

---

### clearedAxes

```
List<E> clearedAxes
```

---

### defaultStyle

```
String defaultStyle
```

---


|  |  |  |
| --- | --- | --- |
| **Overview** \| Package \| Class \| Use \| **Tree** \| **Deprecated** \| **Index** \| **Help** | |  |
| PREV   NEXT | **FRAMES**    **NO FRAMES**     **All Classes** |  |


---
